# A case report of sinoatrial arrest caused by temporal lobe epilepsy in subclinical glioblastoma

**DOI:** 10.1186/s12872-020-01325-3

**Published:** 2020-01-30

**Authors:** Jörg Reifart, Marlene Tschernatsch, Christian W. Hamm, Johannes Sperzel, Andreas Hain

**Affiliations:** 1 Department of Cardiology, Kerckhoff Heart and Thorax Center, Benekestr. 2-8, 61231 Bad Nauheim, Germany; 2 Department of Neurology, Justus-Liebig University Hospital of Giessen and Marburg, Giessen, Germany; 3 Gesundheitszentrum Wetterau, Chaumontplatz 1, Bad Nauheim, Germany; 4 Department of Cardiology, Justus-Liebig University Hospital of Giessen and Marburg, Giessen, Germany

**Keywords:** Cardiology, Sinoatrial arrest, ECG, Glioblastoma, Pacemaker implantation, Bradycardia, Syncope, TLOC

## Abstract

**Background:**

Atrial fibrillation with symptomatic bradycardia, higher grade atrioventricular block, and sinus node disease are all common indications for permanent pacemaker implantation. The most frequent causes of sinus node disease treated with pacemaker implantation involve degenerative structural changes of the sinus node; less often, extrinsic causes (such as damage due to myocardial infarction or heightened parasympathetic nervous system activity) lead to pacemaker implantation.

**Case presentation:**

A 50-year-old patient with syncope and documented sinoatrial arrest was referred. Neurologic exams (including CT and EEG) revealed no pathologies, so a pacemaker was implanted. Postoperatively, syncope occurred again due to a focal seizure during which sinus rhythm transitioned to atrial pacing by the device. Further neurologic testing revealed focal epilepsy. Six months later, stage IV glioblastoma was diagnosed and the patient was treated surgically.

**Conclusion:**

Intracerebral tumors should be considered in the differential diagnosis for patients with unexplained sinoatrial block, as well as in patients with repeat syncope after pacemaker implantation. Cranial MRI could aid the diagnostic workup of such cases.

## Background

Alongside higher grade atrioventricular block and atrial fibrillation with symptomatic bradycardia, one of the leading indications for permanent pacemaker implantation is sinus node disease [1]. Treating symptomatic sinus node disease by permanent pacemaker implantation, preferably dual chamber, is strongly recommended (IB) by the European Society of Cardiology (ESC) guidelines [2].

Functional (secondary) sick sinus syndrome is distinguished from a true (primary) sinus node disorder by its extrinsic causes, such as myocardial infarction, electrolyte disturbances, autonomic dysregulation, or adverse drug reactions. When there is no extrinsic cause, an intrinsic/organic cause is assumed [3].

Bradycardias in association with increased cranial pressure are a well-documented phenomenon [4]. There are some case reports of AV block and asystole occurring with temporal lobe seizures [5].

Here we present a unique case of a sinoatrial block and transient loss of consciousness as the first presenting symptom of subclinical left-sided glioblastoma causing focal temporal lobe epilepsy.

## Case presentation

A 50-year-old otherwise healthy male patient was emergently referred by his primary care physician with recurrent unprovoked syncope which led to multiple hospital visits.

The patient was on beta blocker therapy for supraventricular and ventricular extrasystole, as well as arterial hypertension. There were no other prior medical conditions.

**Fig. 1:**
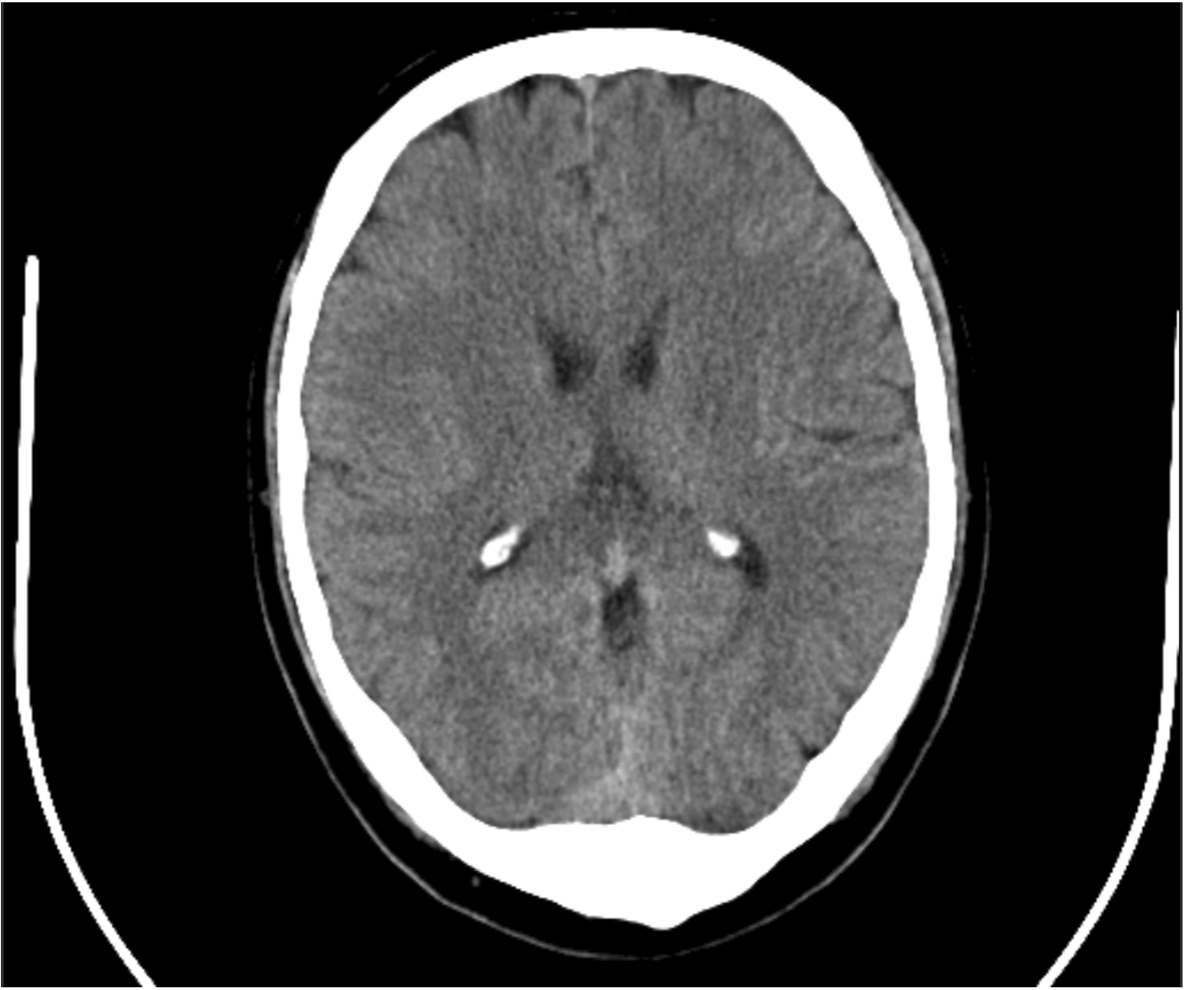
CT before pacemaker implantation

At the time of admission to our hospital, the patient had experienced four episodes of transient loss of consciousness; the last episode lasted 30 s and took place in the office of the primary care physician. The first episode took place after mowing the lawn and caused a minor head trauma leading to his admission to the hospital. A head CT after this incident was deemed normal (Fig. 1); the physical and neurologic exams, as well as repeat EEGs revealed no pathologies.

There were no syncope provoking factors. Since there had been a recent death in the family causing the patient increased psychosocial stress, psychogenic pseudosyncope was also considered. After each episode of transient loss of consciousness, the patient was fully alert and oriented. The patient reported no family history of sudden cardiac death or other cardiovascular diseases.

**Fig. 2:**
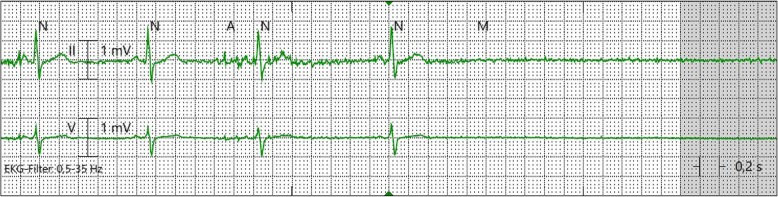
Sinoatrial block

In 24-h Holter monitoring ordered by the primary care physician, a 6 s sinoatrial arrest had been documented. Later, in our clinic, asystole of 12 s was documented (Fig. 2). During the episodes, there were no clinical signs of generalized seizure (no involuntary movements, tongue bite, incontinence, postictal confusion).

After ruling out Lyme disease, relevant coronary artery disease, a structural heart disease, and pulmonary embolism, a dual chamber magnetic resonance (MR) conditional pacemaker was implanted.

**Fig. 3:**
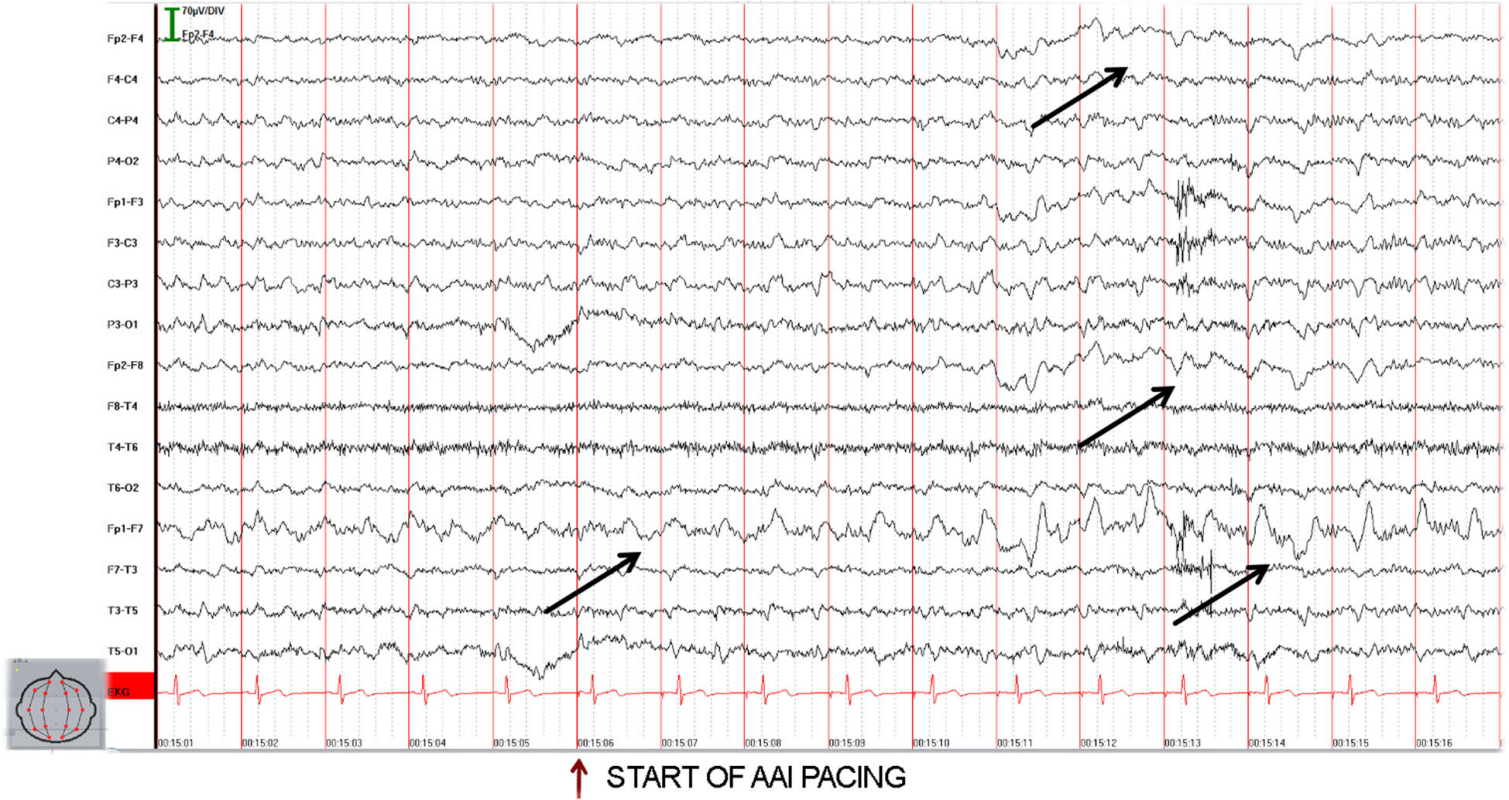
Seizure activity during which pacing occurs – arrows mark beginning of characteristic EEG activity (rhythmic delta-waves, starting left frontal (Fp1-F7) and later lateralizing to the right side)

On the first postoperative day, the patient suffered another transient loss of consciousness. During this episode we observed sinus rhythm transitioning to atrial pacing by the device without a pause on telemetric monitoring. This event prompted a neurological consultation. Again, the clinical neurological exams showed no pathological findings; however during EEG, a hyperventilation provocation test triggered a focal seizure (Fig. 3). Therapy with antiepileptic medication was initiated; at this time the patient’s medication list consisted of an angiotensin-converting-enzyme (ACE) inhibitor because of arterial hypertension, and both acetylsalicylic acid and a statin due to coronary artery sclerosis. He was transferred to a neurology clinic.

Subsequent neurological exams in the neurology clinic, including EEG testing for photosensitive epilepsy triggering and a hyperventilation test, as well as duplex sonography of extra- and intracranial arteries showed normal results and the patient was discharged without changes to his medications.

**Fig. 4:**
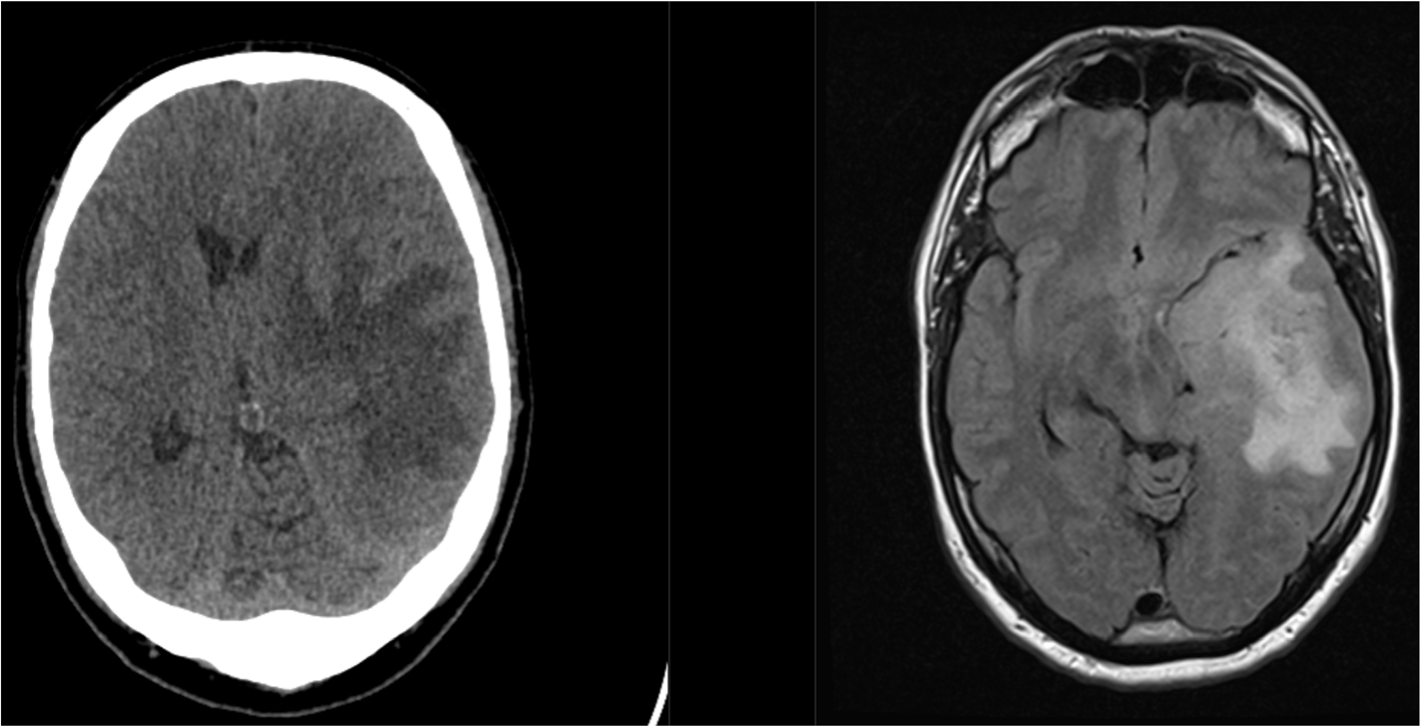
6 months after pacemaker implantation; CT (left) and MRI of newly diagnosed glioblastoma

Six months later, the patient presented with focal seizures, aphasia, and recollection difficulties. Stage IV glioblastoma was diagnosed by CT and MRI and treated surgically (Fig. 4).

## Discussion and conclusions

Focal temporal lobe epilepsy has been linked to syncope and bradycardia by several case reports [5–7]. Here, the patient’s seizure activity may have been secondary to a very early stage glioma. There were no neurological symptoms upon presentation and initial CT showed no evident signs of the later-diagnosed glioblastoma or of intracranial pressure as the cause of the bradycardia (Fig. 1).

As standard workup for syncope in patients without neurological signs does not regularly include provocation EEGs or cranial MRI, unusual cases such as this can easily be missed, which can lead to delay in diagnosis when time is critical.

Unfortunately, pacemaker implantation precludes patients from undergoing a MRI study for 6 weeks [8]. In this case, MRI was only performed when symptoms reoccurred while the patient was on antiepileptic medication, which occurred at 6 months post pacemaker implantation; the lack of earlier MRI performed as soon as possible at 6 weeks post pacemaker implantation certainly prolonged the time to glioblastoma diagnosis for this patient.

In contrast to other reports about the influence of epileptic focal seizures on heart rhythm, here the EEG activity preceded the sudden bradycardia [9] (Fig. 2). Proposed mechanisms could include excessive parasympathetic tone or sympathetic inhibition mediated by vagal nerve stimulation during seizure activity leading to the witnessed extrinsic sinus node dysfunction [10].

In hindsight, the focal seizure in this case did most likely predominantly contribute to the transient loss of consciousness with sinus arrest resulting from neuronal suppression.

Sudden unexpected death in patients with epilepsy is not uncommon. Though often the cause of death remains unclear, arrhythmias such as sinus arrest and bradycardia are thought to contribute to some cases [11].

The workup of syncope can sometimes be challenging. In patients with sinus arrest of unknown origin, intracerebral tumors should be considered in the differential diagnosis. Repeat loss of consciousness after pacemaker implantation should trigger a more intense neurologic workup, possibly including a cranial MRI if the implanted pacemaker is MR conditional or safe.

## Data Availability

Further data and materials may be made available, upon request by the corresponding author, as long as patient anonymity is upheld.

## Abbreviations

AV: Atrioventricular
CT: Computer tomography
EEG: Electroencephalogram
ESC: European society of cardiology
MRI: Magnetic resonance imaging
